# Dengue Virus Activates Membrane TRAIL Relocalization and IFN-α Production by Human Plasmacytoid Dendritic Cells *In Vitro* and *In Vivo*

**DOI:** 10.1371/journal.pntd.0002257

**Published:** 2013-06-06

**Authors:** Mariana Gandini, Christophe Gras, Elzinandes Leal Azeredo, Luzia Maria de Oliveira Pinto, Nikaïa Smith, Philippe Despres, Rivaldo Venâncio da Cunha, Luiz José de Souza, Claire Fernandes Kubelka, Jean-Philippe Herbeuval

**Affiliations:** 1 Laboratório de Imunologia Viral, Instituto Oswaldo Cruz, FIOCRUZ, Rio de Janeiro, Brazil; 2 CNRS UMR 8147, Université Paris Descartes, Paris, France; 3 Chimie et Biologie, Nucléo(s)tides et Immunologie Thérapeutique (CBNIT), CNRS UMR 8601 Université Paris Descartes, Paris, France; 4 Unité des Interactions moléculaires Flavivirus-Hôtes, Institut Pasteur, Paris, France; 5 Departamento de Clínica Medica, FM, Universidade Federal do Mato Grosso do Sul, Campo Grande, Brazil; 6 Centro de Referencia em Dengue, Campos de Goytacases, Brazil; Institute of Tropical Medicine (NEKKEN), Japan

## Abstract

**Background:**

Dengue displays a broad spectrum of clinical manifestations that may vary from asymptomatic to severe and even fatal features. Plasma leakage/hemorrhages can be caused by a cytokine storm induced by monocytes and dendritic cells during dengue virus (DENV) replication. Plasmacytoid dendritic cells (pDCs) are innate immune cells and in response to virus exposure secrete IFN-α and express membrane TRAIL (mTRAIL). We aimed to characterize pDC activation in dengue patients and their function under DENV-2 stimulation in vitro.

**Methods & Findings:**

Flow cytometry analysis (FCA) revealed that pDCs of mild dengue patients exhibit significantly higher frequencies of mTRAIL compared to severe cases or healthy controls. Plasma levels of IFN-α and soluble TRAIL are increased in mild compared to severe dengue patients, positively correlating with pDC activation. FCA experiments showed that *in vitro* exposure to DENV-2 induced mTRAIL expression on pDC. Furthermore, three dimension microscopy highlighted that TRAIL was relocalized from intracellular compartment to plasma membrane. Chloroquine treatment inhibited DENV-2-induced mTRAIL relocalization and IFN-α production by pDC. Endosomal viral degradation blockade by chloroquine allowed viral antigens detection inside pDCs. All those data are in favor of endocytosis pathway activation by DENV-2 in pDC. Coculture of pDC/DENV-2-infected monocytes revealed a dramatic decrease of antigen detection by FCA. This viral antigens reduction in monocytes was also observed after exogenous IFN-α treatment. Thus, pDC effect on viral load reduction was mainly dependent on IFN-α production

**Conclusions:**

This investigation characterizes, during DENV-2 infection, activation of pDCs *in vivo* and their antiviral role *in vitro*. Thus, we propose TRAIL-expressing pDCs may have an important role in the outcome of disease.

## Author Summary

Dengue is an important endemic tropical disease to which there are no specific therapeutics or approved vaccines. Currently several aspects of pathophysiology remain incompletely understood. A crucial cellular population for viral infections, the plasmacytoid dendritic cells (pDCs) was analyzed in this study. The authors found an *in vivo* association between the activation state of pDCs and the disease outcome. Membrane TNF-related apoptosis inducing ligand (TRAIL) expressing pDCs, representing activated pDCs, were found in higher frequency in milder cases of dengue than severe cases or healthy individuals. Detection of antiviral cytokine interferon-alpha (IFN-α) and soluble TRAIL positively correlated with pDC activation. Dengue virus (DENV) serotype-2 was able to directly activate pDCs *in vitro*. Under DENV stimulation TRAIL was relocalized from intracellular to pDC plasma membrane and IFN-α was highly produced. The authors suggest an endocytosis-dependent pathway for DENV-induced pDC activation. It is also highlighted here a role for exogenous IFN-α and pDCs in reducing viral replication in monocytes, one of DENV main target cells. These findings may contribute in the future to the establishment of good prognostic immune responses together with clinical manifestations/warning signs.

## Introduction

Dengue is the most important arthropod-borne emerging viral disease in tropical countries due to its high morbidity and risk of mortality [1]. For example, in Brazil, dengue is a major public health problem and about two million cases were reported during 2010–2012 [2]. Dengue virus (DENV) is a single-stranded RNA virus belonging to genus *Flavivirus* [3], [4]. All DENV serotypes (DENV-1 to -4) may induce a broad spectrum of clinical manifestations from asymptomatic to severe clinical features, characterized by hemorrhagic manifestations and a shock syndrome [5], [6], [7]. High viral load may cause an exacerbated cytokine production that plays a key role in the generation of important physiopathological processes [8], [9]. Human monocytes/macrophages and dendritic cells are susceptible to viral replication [10], [11], [12], [13] and can release soluble mediators involved in vascular permeability and plasma leakage besides coagulation disorders [14], [15], [16], [17].

Dendritic cells link innate and adaptive immunity and play a key role in shaping effective immune responses. Two major subpopulations are described: myeloid or conventional dendritic cells (cDCs) and plasmacytoid dendritic cells (pDCs) [18], [19], [20], [21]. In contrast to cDCs, pDC are not found in homeostatic tissues but mainly in circulating blood and in lymphoid tissues [21], [22], [23]. Despite being rare cells, pDCs produce up to 1,000-fold more IFN-α than other cell types in response to virus exposure [24]. Viral activation of pDCs can be regulated by either one of the two Toll-like receptors (TLR), TLR-7 or TLR-9 [25], which are considered to be the pattern recognition receptors (PRR) for RNA [26] and DNA [27], respectively. It has been shown that cDC are efficiently infected by DENV and that viral replication blocked cDC maturation [28], [29]. However, unlike cDCs, it has been reported that pDCs are not supporting productive DENV replication [30]. Indeed, DENV can activate pDCs through cell endosomal activity and TLR-7 pathway [31]. Furthermore, dengue-infected patients had impaired pDC activation features. Indeed, absolute numbers of blood pDC were decreased [32], [33] and low levels of serum IFN-α [34] were reported.

TNF-related apoptosis-inducing ligand (TRAIL) is a pro-apoptotic molecule, which induces death of cells that express its death receptors (DR), DR4 and DR5 [35], [36]. Furthermore, IFN-α regulates TRAIL expression by several cell types [37]. Soluble or membrane TRAIL mediates apoptosis on cells that are selectively expressing DR4 and DR5, mainly killing virus-infected cells and leaving intact normal cells [38], [39]. Additionally an antiviral role was proposed for TRAIL. DENV-infected monocytes and dendritic cells display reduced viral replication when TRAIL is exogenously administered [40]. Soluble TRAIL (sTRAIL) was found in sera from dengue patients [41], but mTRAIL role and expression by DENV-2 exposed pDC to has not been investigated yet.

In this report we studied pDC activation by DENV and its consequences on viral infection. The clinical study showed that during acute phase of DF, pDCs are activated characterized by TRAIL and IFN-α markers. Indeed, the more pDC are activated the less the disease is severe. We found that DENV-2 efficiently activated TRAIL expression and IFN-α production by pDC. The microscopy study revealed that TRAIL was intracellularly stocked in resting pDC and was relocalized to plasma membrane when pDC were exposed to DENV-2. Furthermore, we showed that pDC could decrease DENV infection in monocytes mainly due to the effects of IFN-α produced. Thus pDC activation constitutes a host defense against DENV-2 infection strongly suggesting that these cells are likely beneficiating the disease outcome.

## Materials and Methods

### Ethics statement

Experimental procedures with human blood have been approved by Necker Hospital Ethical Committees for human research and were done according to the European Union guidelines and the Declaration of Helsinki. Procedures were also approved by the ethical committee at Instituto de Pesquisas Clinicas Evandro Chagas, FIOCRUZ (CAAE 3723.0.000.009-08). All patients were informed of procedures and gave written consent.

### Patient and blood samples

Blood from HIV-1-seronegative blood bank donors was obtained anonymously from “Etablissement Français du Sang” (convention # 07/CABANEL/106), Paris, France. Forty three patients with confirmed dengue fever (Table 1) from two Brazilian Health Centers at Campo Grande, MS and Campos de Goytacases, RJ, Brazil were studied. All patients presented clinical diagnosis of dengue infection.

**Table 1:** Demographic information about the study population with dengue fever (DF)^1^.

Characteristics	DF ± WS		Severe DF^2^	
		*(N)*		*(N)* 3
Age (median years, 25–75%)	43, 26–58	*(33)*	42, 24–50	*(10)*
Sex (M:F; patient number)	14:19		5:5	
Fever	87%	(*31)*	90%	*(10)*
Hospitalization	52%	(*31*)	100%	*(8)*
Hemorrhagic manifestations (mucosal)^4^	16%	*(31)*	30%	*(10)*
Constant vomits	8%	*(25)*	50%	*(6)*
Persistent abdominal pain	8%	*(25)*	60%	*(5)*
Hypotension^5^	4%	*(26)*	25%	*(8)*
Effusions^6^	0%	*(33)*	40%	*(10)*
Platelet counts (×10^3^/mm^3^)^7^	172±37	*(33)*	40±12	*(9)*
Thrombocytopenia (<50.000×10^3^/mm^3^)	18%	*(33)*	78%	*(9)*
Hematocrit	41±1%	*(30)*	42±2%	*(9)*
Hemoconcentration^8^	33%	*(30)*	56%	*(9)*
Previous dengue (IgG positive)	79%	*(30)*	100%	*(8)*
Rapid hematocrit increase and platelet decrease	13%	*(31)*	60%	*(10)*
Leukocyte counts (×10^3^/mm^3^)^7^	4028±522	*(27)*	3818±571	*(8)*
ALT (U)^7^	52±10	*(23)*	2784±2685	*(8)*
AST (U)^7^	73±16	*(23)*	670±611	*(9)*

1 Study population with 43 patients.

2 **DF ± WS** dengue fever without or with warning signs; **Severe DF**, dengue fever with severe clinical manifestations according to WHO criteria [43].

3 Number of patients with the available information during hospitalization.

4 Hemorrhagic manifestations (epistaxis, gengivorrhagia, metrorrhagia, bleeding after coughing).

5 Postural hypotension with decrease in systolic arterial pressure in 20 mmHg in supine position or systolic arterial pressure <90 mm Hg.

6 Pleural, pericardial effusion or ascites.

7 Average ± standard error from minimal recorded platelet, leukocyte/maximal hematocrit counts/ALT or AST values.

8 Elevated hematocrit (20% during course of illness and recovery; or >45%, men and >41%, women).

### Criteria for dengue fever severity and laboratorial diagnosis

Dengue fever was considered mild when no warning signs (WS) or severe clinical manifestations were observed as follows. Dengue fever with WS was considered if patients presented any of the following warnings: (1) abdominal pain or tenderness; (2) persistent vomiting; (3) Clinical fluid accumulation; (4) mucosal bleeding; (5) lethargy; (6) liver enlargement more than 2 cm associated to laboratory parameters as increase in hematocrit (HCT) concurrent with rapid decrease in platelet counts (hemoconcentration or significant increase in hematocrit together with platelet counts bellow 50,000/mm^3^). Severe DF was considered if patient displayed fever of 2–7 days plus any of the following: (1) Evidence of plasma leakage, such as high or progressively rising hematocrit evidenced by hemoconcentration; pleural effusions or ascites; circulatory compromise or shock (tachycardia, cold and clammy extremities, capillary refill time greater than three seconds, weak or undetectable pulse, narrow pulse pressure or, in late shock, unrecordable blood pressure); (2) Significant (internal) bleeding. [42], [43]. Dengue virus infection was confirmed either by anti-dengue-IgM ELISA, serotype specific reverse transcription-polymerase chain reaction (RT-PCR) or by virus isolation as described earlier [44]. Predominant serotypes was Dengue-2 identified in DF±WS (N = 10) and Severe DF (N = 3) but Dengue-1 was also identified in DF±WS patients (N = 6).

### Virus strain and viral stock

Dengue virus type 2 (strain Thailand/16681/1984) [45] was used for virus stock preparation as described elsewhere [46]. Briefly, *Aedes albopictus* cell clone C6/36 (CRL-1660, ATCC) were maintained at 28°C in Dulbecco's modified Eagle Medium (Gibco/Life Technologies, Foster City, CA, USA) with sodium bicarbonate (Sigma-Aldrich, St. Louis, MO, USA) and supplemented with 5% fetal bovine serum (Hyclone, Logan, UT, USA), 1% penicillin-streptomycin-glutamine (Gibco), 0,5% non-essential amino acids (Gibco) and 10% tryptose phosphate broth (Sigma). C6/36 cell monolayers were infected with DENV-2 and cell culture supernatants were harvested 8 days later when cytopathic effect was observed. A purified DENV-2 stock was obtained by ultracentrifugation at 100,000 g for 1 h and set to a final volume 20 times smaller than initial (see also Fig. S1) [47], [48]. Titration was performed in C6/36 cells using a standard TCID_50_ (50% tissue culture infective dose) assay as described elsewhere [49]. Uninfected flasks were maintained, also purified and used as negative control (MOCK). Infectivity of ultracentrifuged virus inoculum (UC) was comparable with the original C6/36 supernatant (SNDT) because infection rates obtained with the dilution 1/100 (UC) is similar to the dilution 1/5 (SNDT) as shown in Fig. S1.

### Human cell isolation

Cryopreserved peripheral blood mononuclear cells (PBMC) from patients or healthy donors were obtained from density gradient centrifugation of heparinized blood with lymphocyte separation medium (StemCell Technologies, Grenoble, FR). *In vitro* experiments were performed using fresh PBMC, which were obtained from blood bank donors and isolated as mentioned above. PDCs and monocytes were purified using Human plasmacytoid DC Negative Isolation Kit and Human CD14^+^ monocytes Isolation Kit, respectively (StemCell Technologies). Cells were cultured in RPMI 1640 (Invitrogen, Gaithersburg, MD, USA) containing 10% fetal bovine serum (Hyclone) and 1% penicillin-streptomycin-glutamine (Gibco) at 37°C in a humidified 5% CO_2_ chamber according to protocol.

### PDC stimulation and coculture with monocytes

Freshly purified pDCs were cultured with DENV-2 at approximately MOI 4 to 20, mock for 18 hours (overnight). Chloroquine (Sigma-Aldrich) was used at 5 µM/well and added before viral stimulation. Cells were harvested and assessed for pDC cell markers and membrane TRAIL expression or plated on coated slides for 3D microscopy. Supernatant was stored at −70°C for cytokine detection. Monocyte infection was performed as already described [46]. Briefly, freshly isolated monocytes were plated overnight followed by infection with DENV-2 at MOI 10, mock or not infected for 48 hours. Soluble human recombinant IFN-α (PBL International, Piscataway, NJ, USA) was added 18 hours before viral infection at 100 IU/mL. For autologous coculture assay, monocytes were cultured overnight in media, meanwhile pDCs were chloroquine-treated or not and then stimulated overnight with CpG A 2216 (InvivoGen, San Diego, CA, USA) at 5 µM or DENV-2 at MOI 20, or not stimulated. Monocytes were then infected with DENV-2 (MOI 10) and pDCs were added at ratio 1∶5 pDC/monocytes as explained in Fig. S2. Cells were harvested and assessed for intracellular DENV antigens.

### Flow cytometry

Antibodies for fluorescein isothiocyanate (FITC)-conjugated anti-CD123 or BDCA-2 (Miltenyi Biotec, Auburn, CA), Phycoerythrin (PE)- conjugated CD11c (IOTest/Beckman Coulter, Marseille, FR), Allophycocyanin (APC)-conjugated anti-BDCA-4 (Miltenyi Biotec) and Allophycocyanin-Cy7 (APC-Cy7)-conjugated anti-CD14 (BD Biosciences, San Jose, CA), Vioblue-conjugated anti-CD4 (Miltenyi Biotec), V500 anti-CD3 (BD Biosciences) or with appropriate isotype-matched control antibodies (at 5 mg/mL each) in PBS containing 2% fetal bovine serum (Hyclone) and 2 mM EDTA (Gibco). Human PBMCs or isolated monocytes/pDCs were incubated for 20 min at 4°C with antibody cocktails. Cells were washed twice in ice-cold PBS and flow cytometry acquisition was performed on FACSCanto 7 colors or FACS Aria 13 colors flow cytometers using FACSDiva software (BD Biosciences). CD3^−^ CD4^+^ CD14^−^ CD123^+^/BDCA-2^+^ BDCA-4^+^ gated cells were then tested for the expression of surface markers using PE-labeled anti-TRAIL (BD Biosciences). Mosquito C6/36 cell line monolayers were washed with PBS-1% bovine serum albumin (Sigma) and incubated for 60 min at 4°C with purified anti-DENV-complex (Millipore, Billerica, MA, USA) then 30 min with goat anti-mouse Alexafluor647 (Molecular Probes/Life Technologies) and fixed. Intracellular antigen staining for C6/36 or cocultures was performed using 2% paraformaldehyde (Sigma) followed by antibodies staining steps with 0,1% saponin (Sigma) buffer. Cells were analyzed by C6 Cytometer (Accuri/BD Biosciences). FlowJo software (Treestar, Ashland, OR, USA) was used to analyze flow cytometry data.

### Three dimension (3D) microscopy and immunofluorescence

Cells were plated on poly-L-lysine (Sigma)-coated slides and then fixed in 4% paraformaldehyde (Sigma), quenched with 0.1 M glycine (Sigma). Cells were blocked and incubated in permeabilizing buffer containing 0.1% saponin (Sigma) with mouse anti-TRAIL (clone RIK-2, eBioscience, San Diego, CA) or mouse anti-DENV (clone D3-2H2-9-21, Millipore). TRAIL and DENV staining were revealed using a secondary donkey anti-mouse IgG-Cy3 (Jackson ImmunoResearch, West Grove, PA, USA). Nucleus was stained using DAPI (Molecular Probes/Life Technologies). Mounted slides were scanned with a Nikon Eclipse 90i Upright microscope (Nikon Instruments Europe, Badhoevedorp, The Netherlands) using a 100× Plan Apo VC piezo objective (NA 1.4) and Chroma bloc filters (ET-DAPI, ET-Cy3) and were subsequently deconvoluted with a Meinel algorithm and 8 iterations and analyzed using Metamorph (MDS Analytical Technologies, Winnersh, UK). Overlays were: TRAIL or DENV/DAPI/Trans. ImageJ (NIH, Bethesda, MD, USA) plugin 3D interactive surface plot was used on overlay stack on pDC stained with TRAIL or DENV/DAPI. Quantity of TRAIL and DENV-2 were determined using the measure and label plugin (ImageJ). C6/36 mosquito cell line were plated on slides and fixed with cold acetone. Mosquito cells were stained with mouse anti-DENV complex (Millipore) in PBS-1%bovine serum albumin (Sigma), washed twice with PBS. DENV E protein was revealed with goat anti-mouse Alexafluor488 (Molecular Probes/Life Technologies). Slides were mounted with ProLong Gold with DAPI (Molecular Probes/Life Technologies) and visualized at Evosfl Microscope (AMG, Bothell, WA, USA).

### Cytokine detection

Supernatants of pDCs/monocytes or cocultures in presence of DENV-2 or negative controls as well as acute phase plasma from dengue patients were tested for multispecies soluble IFN-α by ELISA (PBL International) according to the manufacturer's instructions. Plasma samples were also tested for soluble TRAIL by ELISA (R&D Systems, Minneapolis, MN, USA)

### Statistical analysis

Experiments were repeated at least four times. P values (*P*) were determined using a two-tailed Student's *t* test for *in vitro* data and nonparametric Mann-Whitney test for patient data. *P*<0.05 was considered statistically significant. Univariate distributions of flow cytometric data were performed by probability binning, in 300 bins using FlowJo software [50].

## Results

### Dengue patients differentially exhibit TRAIL^+^ pDCs, soluble IFN-α and TRAIL levels

**Figure 1:**
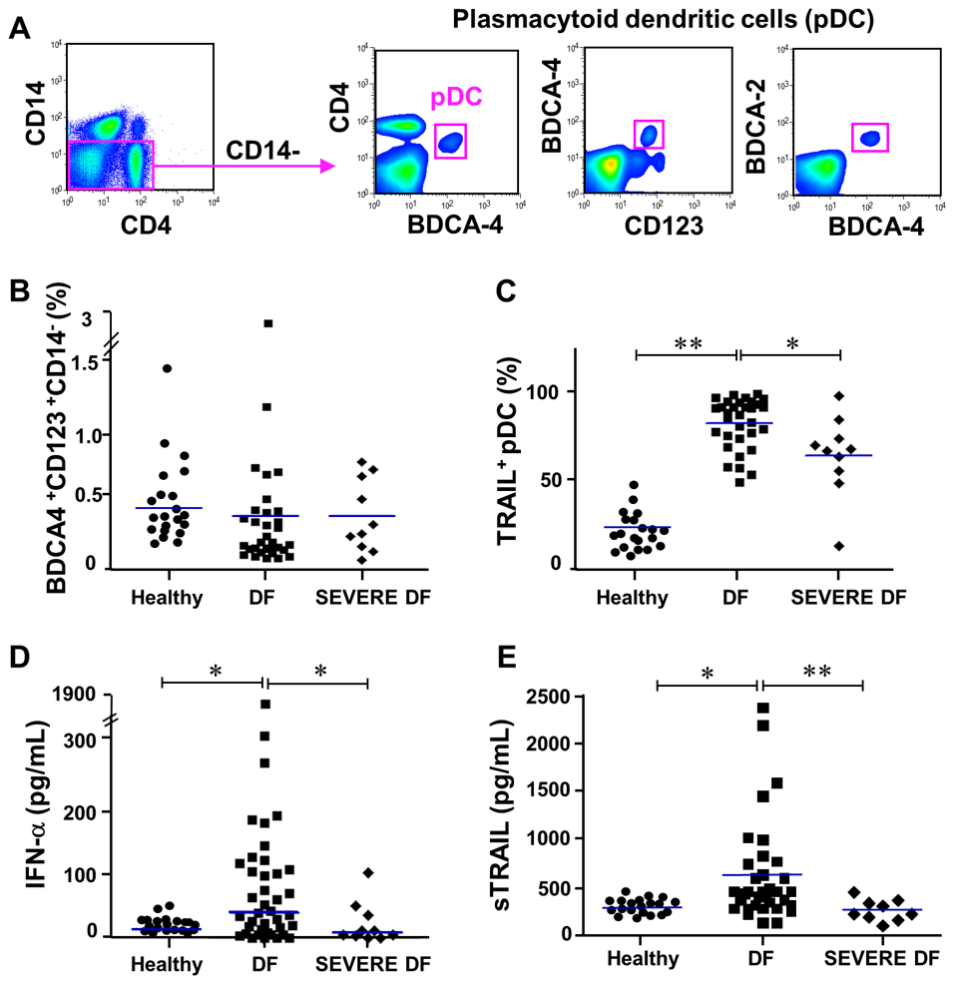
TRAIL and IFN-α expression on dengue fever (DF) patients. PBMCs from acute dengue fever patients were analyzed for mTRAIL expression on pDCs gated as CD14^−^, CD3^−^, CD4^+^, CD123^+^ and BDCA-4^+^ (**A**). (**B**) Blood pDC percentages and (**C**) mTRAIL expressing pDCs were assessed for healthy donors, DF and severe DF patients. (**D**) IFN-α and (**E**) Soluble TRAIL were analyzed by ELISA in plasma samples from healthy donors, DF and severe DF patients. Each dot represents one individual and median values are shown as blue bars. Data values were submitted to Mann-Whitney statistical test in which * *p*<0.05 and **p<0.005.

We studied a cohort of DENV infected patients and classified them regarding the severity of the disease. Detailed demographic, clinical, and laboratorial data from dengue patients are summarized in Table 1. From 43 patients enrolled, 10 were classified as severe DF and the remaining as DF including those with warning signs for severity (WS), according to latest WHO classification [42], [43]. In order to explore pDC activation by DENV infection, we first characterized the CD4^+^/CD14^−^/BDCA-2/4^+^/CD123^+^ pDC frequency/profile in 40 patients compared to 20 healthy controls (figure 1A).

As described by others [51], pDC frequencies in healthy individuals range from 0.2% to 0.8% of peripheral blood mononuclear cells (PBMCs). We observed no significant differences in pDC frequencies among healthy donors, DF±WS patients or Severe DF patients (figure 1B). We then observed that mTRAIL expression on pDC was increased in DF±WS patients compared to healthy controls or severe DF cases (figure 1C). Therefore, pDCs become activated in dengue patients with regard to mTRAIL expression.

Although pDCs are not the only IFN-α producers, activated pDCs can support a 1000-fold greater production of this factor than other cell types. We next sought a correlation of IFN-α with severity. Soluble IFN-α level in plasma samples from the studied population was determined by ELISA. Similarly to TRAIL^+^ pDC frequency, we found that DF patients exhibit higher levels of IFN-α compared to healthy controls or Severe DF patients (figure 1D). Indeed, we found a positive correlation between IFN-α levels and TRAIL^+^ pDCs (Spearman r = 0.36, *p*<0.05). To further determine the IFN-α role, we quantified soluble TRAIL (sTRAIL) levels that is produced by immune cells and is induced by type I IFN. Similarly to previous data, DF±WS patients displayed elevated sTRAIL in contrast to healthy controls or severe DF patients (figure 1E). Moreover, a strong positive correlation between TRAIL^+^ pDCs and sTRAIL was determined (Spearman r = 0.60 *p*<0.005). PDC activation during dengue fever, elevated IFN-α and TRAIL levels is therefore associated with mild dengue fever.

### DENV-2 activates pDC leading to TRAIL display at cell surface and IFN-α secretion

**Figure 2:**
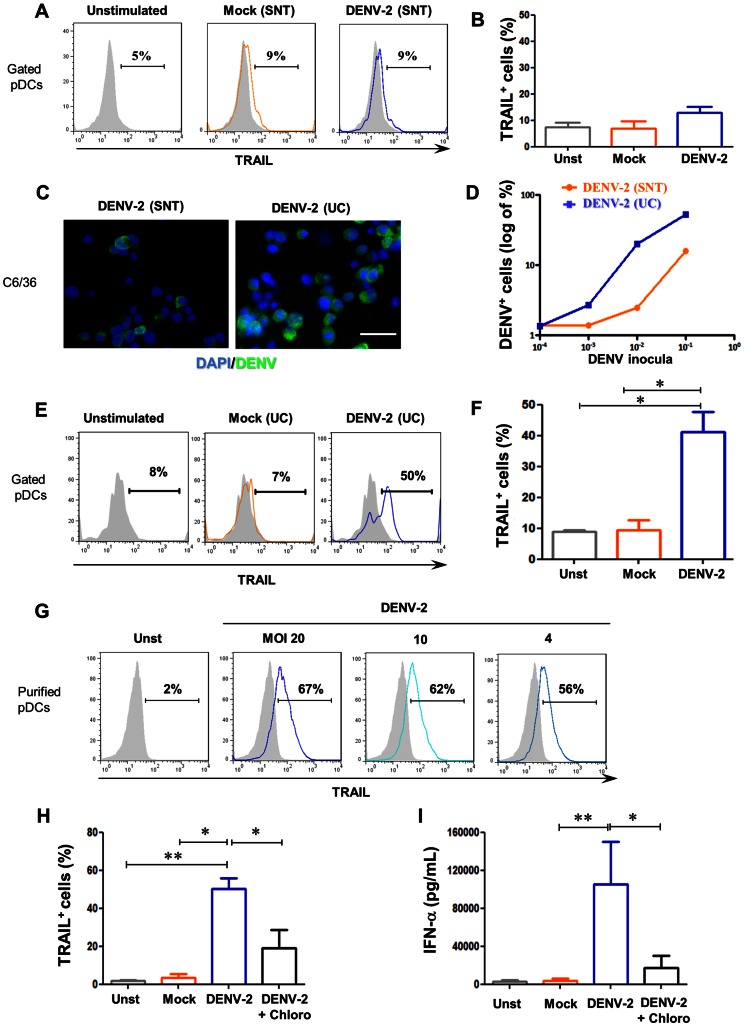
Purified DENV-2-induced *in vitro* mTRAIL expression and IFN-α production by purified plasmacytoid dendritic cells. PBMCs from healthy donors were stimulated overnight with DENV-2, mock or none (unstimulated). (**A**) mTRAIL expression profile on pDCs gated from PBMCs (overlay) and (**B**) mTRAIL positive pDCs for three donors induced by mock SNT (orange) or DENV-2 SNT (blue) using unstimulated (grey fill) pDCs as negative control. DENV positive C6/36 cells infected for 48 h with supernatant of DENV-2-infected C6/36 cells (DENV-2 SNT) or ultracentrifuged DENV-2 SNT (DENV-2 UC) as described in M&M and figure S1. (**C**) DENV antigens/AlexaFluor488 (green) and nucleus/DAPI (blue) of C6/36 cells infected with DENV-2 SNT (left) and UC (right) at the same inocula dilution (10^−3^). (**D**) DENV positive C6/36 cells by flow cytometry in which cells were infected with SNT (orange) or UC (blue) DENV-2 inocula at different dilutions. PBMCs from healthy donors were stimulated overnight with DENV-2 UC, mock UC or none (unstimulated). (**E**) mTRAIL expression profile on pDCs gated from PBMCs (overlay) and (**F**) mTRAIL positive pDCs for four donors induced by mock UC (orange) or DENV-2 UC (blue) using unstimulated (grey fill) pDCs as negative control. Freshly purified pDCs were stimulated overnight with DENV-2 UC, mock UC or not (unstimulated). (**G**) TRAIL expression induced by different MOIs of DENV-2 UC (blue) using unstimulated cells (grey) as negative control. (**H**) Purified pDCs positive for mTRAIL expression and (**I**) IFN-α secretion by unstimulated (grey), mock UC (orange), DENV-2-UC-stimulated pDCs pre-treated (black) or not (blue) with chloroquine, for four donors. Values were submitted to paired t test in which * p<0.05 and ** p<0.005.

PDC activation by DENV-2 was shown to occur by TLR-7 stimulation after endocytosis [31] and this pathway was crucial for IKpDC transformation by HTLV-1 [48]. To assess pDC activation by DENV-2, peripheral blood mononuclear cells (PBMCs) from healthy donors were stimulated overnight with virus. Initially, we observed that DENV-2 from mosquito cell line supernatant (SNT) promoted a trend, however not statistically significant, in TRAIL detection on pDC surface after viral stimulation in PBMCs, compared to unstimulated or mock-stimulated pDCs (figure 2A and B). Thereafter, an ultracentrifugation of DENV-2 viral stock was performed in order to concentrate viral particles, increasing MOI (figure S1). The DENV-2 infectivity was assayed for both viral stocks by infecting the mosquito cell line C6/36 and comparing them in serial dilutions. Viral antigens were detected inside cells inoculated with concentrated DENV-2 (UC) as early as 48 hours and at higher frequencies than the non-concentrated supernatant indicating that the concentrated virus had enhanced replication rates and it was intracellularly present as detected by immunofluorescence microscopy and flow cytometry (figure 2C and D). This viral stock (DENV-2 UC) was therefore adopted for assessing DENV-2 induced pDC activation in all experiments described in the present work.

Therefore, using purified virus in PBMC cultures we observed an increase of mTRAIL detection (figure 2E and F) in 41%±6% of pDCs (CD4^+^ CD14^−^ BDCA4^+^ CD123^+^) compared with less than 10% TRAIL^+^ pDCs on mock or unstimulated conditions (*p*<0.05). To exclude pDC bystander activation and to confirm that DENV-2 is directly inducing mTRAIL on pDCs, we assayed purified pDC for TRAIL and IFN-α production. Purified pDC were exposed to different multiplicities of infection (MOI) for DENV-2 and we observed an increased inoculum-dependency of mTRAIL detection by virus-activated pDCs (figure 2G). The mTRAIL displayed on cell surface was mostly blocked when pDCs were pre-treated with chloroquine, an endosomal blocker of TLR activation, supporting the concept of an endocytosis-TLR-dependent TRAIL activation (figure 2H). To further characterize DENV-2-induced activation of pDCs, we measured IFN-α production in purified pDC cultures supernatants. DENV-2-stimulated pDCs produced approximately 10,000-fold more IFN-α than mock-treated or not stimulated pDCs. Chloroquine pre-incubation abrogated most DENV-2-induced IFN-α production (figure 2I). These results confirm that DENV-2 is able to activate pDCs *in vitro* through endocytosis pathway, responding by TRAIL expression and IFN-α production.

### DENV-2 and TRAIL location within pDCs by 3-dimension microscopy

**Figure 3:**
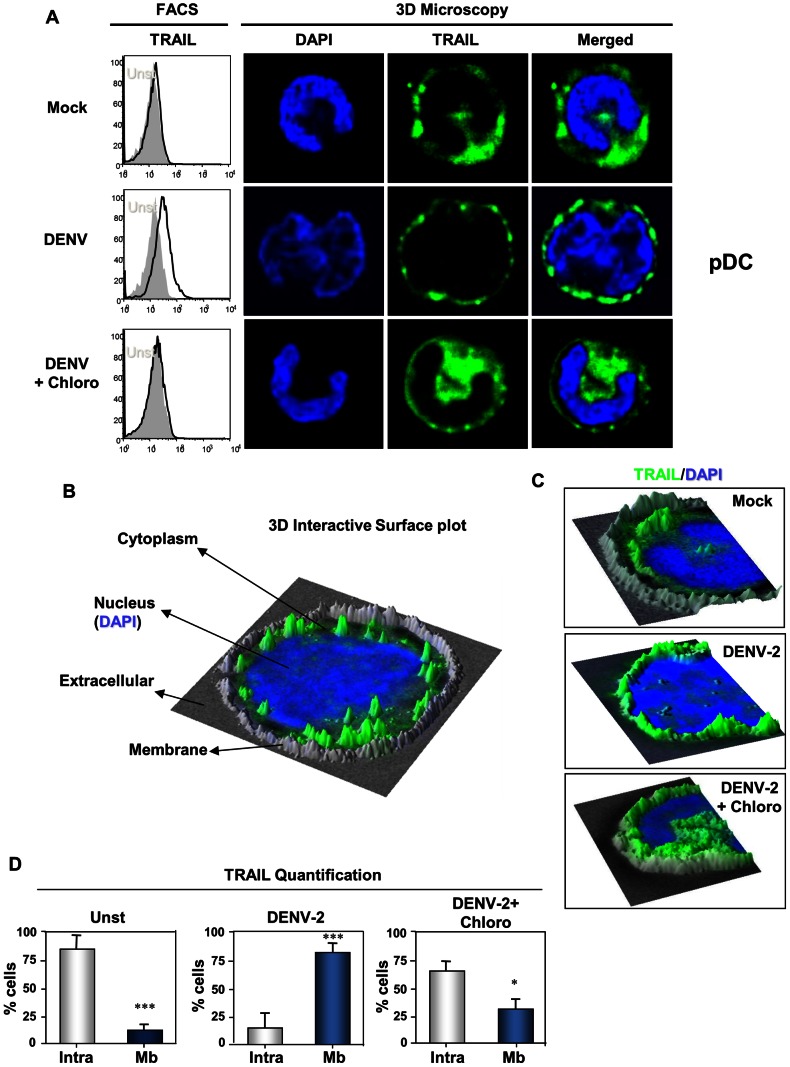
TRAIL localization in DENV-2-activated pDCs by 3D microscopy analysis. Freshly purified pDCs stimulated with DENV-2 pre-treated or not with chloroquine (Chloro), or mock infected. TRAIL expression was analyzed by flow cytometry or by a 3D microscope. (**A**) Membrane TRAIL flow cytometry profiles (left column) on pDC stimulated by different stimuli -mock, DENV-2 or DENV-2+Chloro overlaid by unstimulated (grey). Microscopic images from pDC cultured with the mock, DENV-2 or DENV-2+Chloro showing DAPI-colored nucleus, TRAIL staining (green) and overlay. (**B**) 3D interactive surface plots analysis of 3D microscopic image. Overlay of nucleus (blue), TRAIL (green) and phase contrast (grey) as seen in (**C**) for different stimuli: DENV-2-stimulated pDC (DENV-2) exhibits membrane TRAIL localization in contrast to DENV-2+Chloro or unstimulated, where TRAIL is detected only intracellularly. (**D**) Percentage of pDCs expressing intracellular TRAIL only (Intra) or on the membrane (Mb) is shown as percentage of total analyzed cells. Values were submitted to paired t test in which * p<0.05 and *** p<0.0005.

To better characterize DENV-2-activated pDCs, we analyzed them by 3-dimension (3D) microscopy. Focal plane analysis revealed the presence of intracellular TRAIL expression in unstimulated pDC (figure 3A, upper panels), confirming our cytometry data and our previous study [48]. Images also revealed some ‘peripheral’ TRAIL expression that did not seem to be localized in the cytoplasm but rather on the membrane (figure 3A, middle panels). TRAIL expression profile in DENV-2-stimulated pDC did not seem to differ from unstimulated cells, even if TRAIL appeared to be decreased in the cytoplasm at the expense of “peripheral” TRAIL (figure 3A, middle panels). However, it remained hard to distinguish between intracellular and membrane TRAIL profile expression in both conditions without the use of a membrane marker. The blocking of endosomal acidification by chloroquine use revealed the same profile as mock-stimulated pDCs.

Thus, to better characterize TRAIL localization in pDCs, 3D reconstruction (focal plan, XZ and YZ-stacks) analysis was performed (figure 3B–D). 3D interactive surface plot plugin of ImageJ software combined with phase contrast acquisition allowed us to visualize with precision internal or external localization of TRAIL (membrane delimitation) (figure 3B). This combined analysis clearly showed intracytoplasmic TRAIL repartition of mock stimulated pDC (figure 3B and C upper panel). DENV-2-stimulated pDC (figure 3C, middle panel) mainly harbored membrane TRAIL localization in contrast to the restrictive intracellular TRAIL expression of pDC from mock stimulated cells (figure 3A–B, right panels). The addition of the endocytosis-TLR pathway inhibitor chloroquine induced an intracellular blocking of TRAIL by DENV-2 exposed pDC. Quantification of membrane *vs.* intracellular TRAIL in pDC by 3D microscopy in independent assay sets demonstrates a clear shift from intracellular to membrane TRAIL location under DENV-2 exposure (figure 3D). We observed that almost all mock stimulated pDCs express only intracellular TRAIL. Chloroquine-treatment of DENV-2-stimulated pDC cultures prevented most TRAIL membrane co-localization on pDCs. Considered together, these results demonstrate that DENV-2 induces TRAIL relocalization from intracellular compartment to pDC plasma membrane.

**Figure 4:**
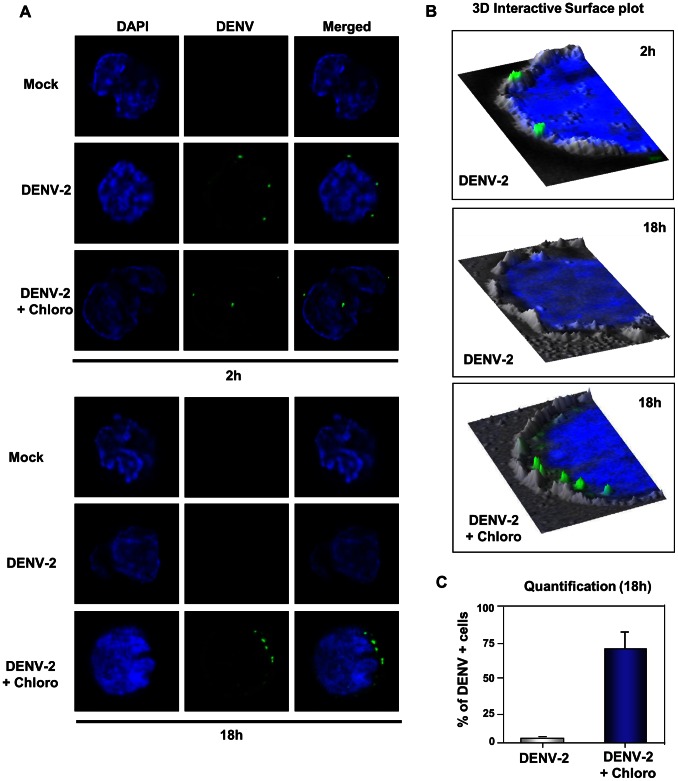
3D microscopy of DENV-2 particles in purified plasmacytoid dendritic cells. Freshly purified pDCs cultured with DENV-2 pre-treated or not with chloroquine (Chloro), or mock infected were stained with anti-DENV (green) and nucleus was colored with DAPI (blue). (**A**) pDC images (nucleus, virus and overlay) for mock, DENV-2 and chloroquine-treated plus DENV-2. Inhibition of endosomal acidification (chloroquine) allowed easier detection of DENV particles (DENV-2+Chloro at 2 h or 18 h stimulation). 2 h pDC incubation with DENV-2 was sufficient to detect viral proteins in contrast to the overnight (18 h) DENV-2-incubated pDCs when no virus was detected. (**B**) pDCs cultured with mock, DENV-2 or DENV-2+chloro were observed by 3D microscope. DENV staining (green) was merged with DAPI (blue)-colored nucleus and with phase contrast (grey). DENV particles were co-localized with pDC cell membrane at 2 h stimulation. Chloroquine allowed DENV-2 detection inside pDCs after 18 h of culture whereas DENV-2 alone did not. Panels shown microscopic images analyzed by 3D interactive surface plot. (**C**) Quantification of PDCs expressing DENV antigens without (DENV-2) and with chloroquine pre-treatment (DENV-2+Chloro) is shown as percentage of total analyzed cells.

We also attempted to detect virus inside pDCs by 3D microscopy. Because virus is rapidly degraded in endosomes by acid-activated proteases, we analyzed DENV-2 localization as early as 2 hours of viral stimulation. Focal plane images revealed that DENV-2 envelope protein was detected in close proximity to pDC periphery. In contrast, DENV-2 seemed to be intracellular in chloroquine treated pDC (figure 4A, upper panel). However, after overnight culture, DENV-2 labeling was exclusively detected in chloroquine-treated cells (figure 4A, lower panel). As described above for TRAIL detection, a 3D interactive surface plot analysis was performed and clearly showed that DENV-2 was co-localized in the cell membrane after 2 hours of stimulation (figure 4B, upper panel). We did not detect any virus in pDCs, suggesting a complete viral degradation within lysosomes either overnight (figure 4B, middle panel) or after a 2 hour-stimulation. However, DENV-2 particles were detected inside chloroquine-treated pDCs, indicating that chloroquine would probably neutralize acid proteases allowing viral antigen detection within most pDCs (figure 4B, lower panel and 4C). Therefore, within the same stimulus, pDCs exhibit TRAIL relocalization at the time point when no virus was detected, supporting our data for endosomal activation of TRAIL pathway.

### DENV-2 infection is impaired in monocytes during coculture with activated pDCs

**Figure 5:**
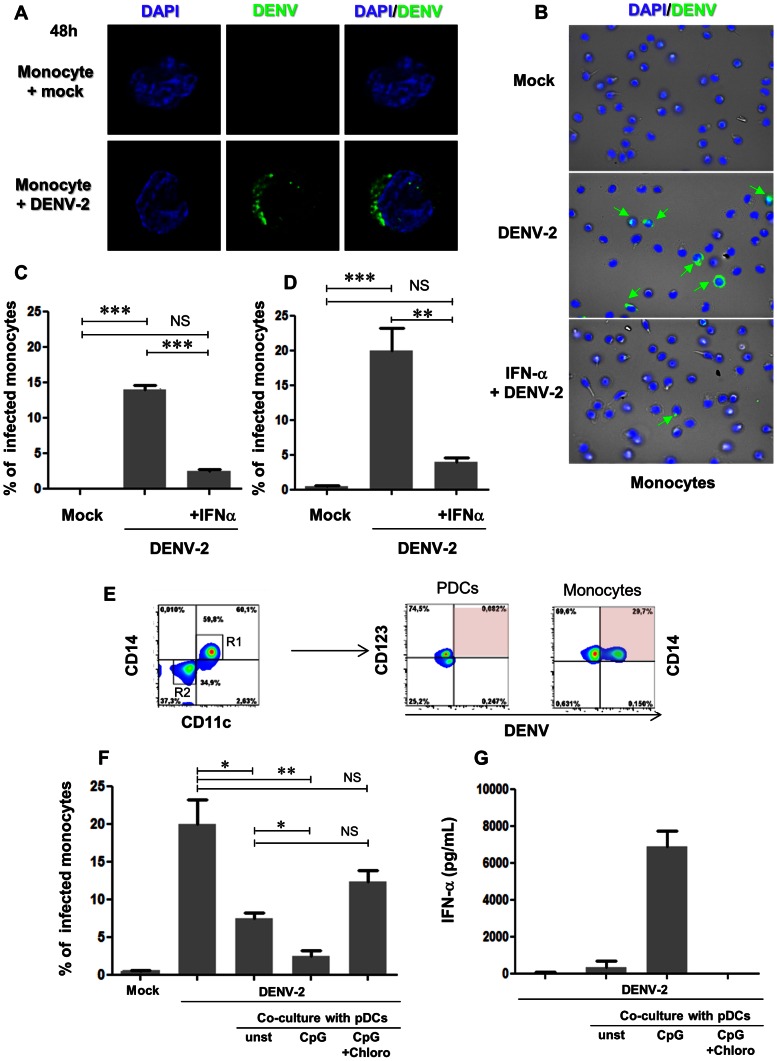
IFN-α treatment and activated pDC coculture in DENV-2-infected monocytes. Freshly purified monocytes were infected with DENV-2 (MOI 10) for 48 hours, pre-treated or not with IFN-α. (**A**) Nucleus/DAPI (blue) and virus (green) of monocytes mock- (upper panels) or DENV-2-infected (lower panels). Viral particles were detected in the cytoplasm only in DENV-2-infected monocytes. (**B**) Nucleus/DAPI (blue), virus (green) and phase contrast (grey) for different stimuli (mock – top, DENV-2 only – middle, IFN-α+DENV-2 – bottom) in monocytes. Green arrows show intracellular detection of DENV particles in DENV-2 infected monocytes, whereas pre-treatment with IFN-α strongly reduced viral antigen detection. Quantification of DENV antigens in mock, DENV-2 and IFN-α pre-treated DENV-2-infected monocytes using microscopy (**C**) or flow cytometry (**D). (E**) Freshly purified monocytes were DENV-2-infected then immediately cocultured with DENV-2-stimulated pDCs. DENV antigen detection in CD14-CD11c-CD123+ pDCs or CD14+CD11c+ monocytes after 48 h of culture. Monocytes were DENV-2-infected then immediately cocultured with CpG-stimulated only (CpG), Chroloquine pre-treated CpG-stimulated (CpG+Chloro) or unstimulated (unst) pDCs (figure S2). Cocultures were analysed 48 h later for viral antigens (**F**) and IFN-α production (**G**). Data represent independent experiments from two different donors and values were submitted to paired t test in which * p<0.05; ** p<0.005 and *** p<0.0005.

Because viral load is considered to be an important factor in dengue severity [8], we next studied the role of pDCs in viral replication. For that purpose, we used primary autologous human monocytes that allow efficient DENV-2 replication in order to assess whether pDC could inhibit viral replication or not. Analysis of purified monocytes infected for 48 hours revealed in 2D microscopy a robust intracellular but not nuclear staining of DENV proteins (figure 5A, lower panel), consistent with flavivirus replication cycle [4]. Considering that pDCs produce high levels of IFN-α upon DENV-2 stimulation, we evaluated its antiviral effect. Monocytes that were pre-treated with IFN-α 24 hours before DENV-2 incubation showed a great reduction in viral antigen detection compared to untreated cells (figure 5B). Quantification by microscopy of DENV-2 positive/negative cells showed that IFN-α treatment reduced by 80% (p<0.001) the number of DENV-2 positive cells (figure 5C) and the same reduction was observed by flow cytometry (figure 5D). We also observed a low production of IFN-α by DENV-infected monocytes and confirmed IFN-α on supernatants of monocytes pre-treated with the cytokine (figure S2). These data are supporting that IFN-α has a restricting antiviral role during DENV infection.

Thus, to determine the potential effect of pDC on DENV infection within monocytes, we cocultured pDCs with infected monocytes. First, we confirmed that viral antigens were only detected in monocytes during cocultures, as only CD14^+^CD11c^+^ DENV-2-infected monocytes display DENV antigens compared to CD14^−^CD11c^−^CD123^+^ DENV-2-stimulated pDCs (figure 5E). To activate pDC in a non-viral way, we stimulated them with the TLR-9 agonist CpG, which was reported to induce IFN-α production and TRAIL expression by pDC [48]. DENV-2 detection on monocytes was significantly diminished when cells were incubated with CpG-stimulated pDCs including supernatants (figure 5F). Importantly, non-pre-activated pDCs also diminished DENV-2 infection in monocytes, although to lesser extent. Chloroquine, which is an inhibitor of TLR-9 pathway, blocked pDC activation and partially restored DENV^+^ cell detection within monocytes. Indeed, IFN-α was highly detected in the cocultures of infected monocytes with CpG-activated pDCs compared to non-pre-activated pDCs (figure 5G). Chloroquine completely blocked IFN-α secretion, suggesting the more IFN-α produced by activated pDCs the less viral antigens are detected. These findings are strongly supportive for an important role for pDC on DENV replication in monocytes.

Finally, we also tested whether IKpDC-mediated apoptosis was involved in reduced DENV-2 detection in monocyte-pDC coculture. Monocytes were infected with DENV-2 or mock and then co-cultured with or without CpG-stimulated with or without pDCs (figure S3). After 48 hours of infection, cultures were collected and stained for AnnexinV and TOPRO3. We observed that the addition of unstimulated pDC to DENV-infected monocytes had no major impact on cell death during co-culture, remarkably, when compared to DENV only. Furthermore, CpG-activated pDCs addition caused an increase survival of monocyte during co-cultures, meanwhile reducing viral antigens. Therefore, we could rule out the killing effect of IKpDCs and once more attribute an antiviral role for IFN-α (and/or TRAIL) in the supernatant.

## Discussion

The present work describes features of pDC activation during DENV-2 infection and discusses its importance for disease outcome. We characterized, for the first time, an activated profile of pDCs from dengue patients using membrane TRAIL expression as a marker. Moreover, we observed that, *in vitro*, activated pDCs exerted an antiviral activity in infected human primary monocytes. Thus, pDCs may contribute to the control of viral clearance and to diminish the severity of the disease.

Upon challenge by viral particles, pDC activation takes place, characterized by upregulation of co-stimulatory markers, and by very high levels of IFN-α secretion [52]. Simultaneously to IFN production, we previously demonstrated that viral-activated pDC also expressed the pro-apoptotic ligand TRAIL on their membrane, which transforms them into IFN-producing Killer pDC (IKpDCs) [48], [53], [54]. For instance, in HIV-1 infection, the number of IKpDCs was correlated to CD4 depletion and disease progression [53]. However, the pDC function depends on the etiology of viral infection. During dengue disease, pDC activation by membrane TRAIL expression was found associated with less severe clinical manifestations. Other studies have also assessed blood pDCs from DENV-infected patients. Reduced absolute numbers of these cells were associated with a poor outcome, because severe cases of dengue disease exhibit a lower number of blood pDCs [32] and low levels of blood pDCs were correlated with high viral loads [33]. Nevertheless, treatment with TLR-3 and -7/8 agonists enhanced pDC activation and reduced viral replication in non-human primate model during DENV infection [55]. Supposedly, a blunted pDC response would allow viral replication to take place. Therefore, we also decided to characterize plasma levels of pDC-related cytokines.

Viral activation of PDC leads to production of IFN-α. Although pDC does not produce sTRAIL, pDC-produced IFN-α leads to production of soluble or membrane bound TRAIL by several cell types including monocytes [56]. Because IFN-α and TRAIL were reported to be antiviral *in vitro* for DENV, we analyzed the soluble levels in dengue patients. Indeed, plasma levels of both factors were statistically correlated with pDC activation in our cohort. Regarding blood cytokine levels in dengue patients, we find discrepancies in literature [9]. Inflammatory cytokines are increased in severe cases compared to mild forms. Even though, IFN-α was reported in DF and DF severe cases [57], Chen *et al.* detected higher IFN-α levels in DF compared to severe cases [34], supporting our data. Soluble TRAIL levels were not associated to severe forms but to febrile period and to primary infections [41]; however, we found a negative association between soluble TRAIL and severity. Because TRAIL is a downstream IFN stimulated gene, reduced IFN-α levels could explain low levels of soluble TRAIL in severe patients. Moreover, a weak type I IFN response in severe DF patients could represent a viral escape pathway. Others have reported that some viruses can evade TLR-induced IFN-α production, by inhibiting pDC function through the binding to BDCA-2, a cell surface molecule that functions as IFN secretion inhibitor [58], [59]. Indeed, BDCA-2 attachment can also abolish TRAIL-mediated cytotoxicity of pDCs [60]. It remains to be investigated whether DENV proteins can downregulate pDC function. This could explain why some patients respond efficiently to DENV infection and show high levels of IFN-α and sTRAIL, while others do not produce sufficient levels of the factors (severe cases). Furthermore, elevated numbers of activated pDCs could, by releasing high levels of IFN-α, protect target cells and activate other innate immunity actors, like Natural Killer cells that are associated with mild DF [61]. Therefore we suggest a protective role of activated pDCs during acute phase of dengue virus infection.

We asked whether pDCs could acquire IKpDC phenotype and have a protective role against DENV infection *in vitro*. DENV-2 induced IFN-α production and TRAIL relocalization from the intracellular compartment (in resting pDC) to pDC membrane (activated pDC) soon after viral exposure, supporting the idea of a rapid response to viruses. A high viral load was necessary to activate pDCs that was only achieved after a concentration procedure using ultracentrifugation protocols [47], [48]. Although purification and ultra-centrifugation protocols may decrease infectious-to-particle ratio [62] our concentrated inoculum displayed improved infectious features. However, we cannot rule out that both non-infectious and infectious particles are activating pDCs in synergism, as it was shown for HIV. Indeed, infectious and AT-2-treated HIV (non-infectious) were both able to activate TLR-7 pathway in pDCs [63]. Apparently, it seems that pDCs need large quantities of virus to be activated or high frequency of viral receptor [64]. HTLV-1 also required high viral loads to activate IKpDCs [48]. Indeed, some discrepancies of IFN-α production by DENV-stimulated pDC are reported that may be result from using low viral loads [65] [66]. Flaviviruses may have acquired intrinsic mechanisms to avoid pattern recognition receptors [67] and consequently pDC activation.

To better elucidate pDC activation by DENV, we studied endocytosis pathway in pDC. Lysosomal acidification was crucial for TRAIL expression and IFN-α production by DENV-2 activated pDC. Another report showed that TLR-7 was the endosomal recognition receptor for DENV-2 by using specific inhibitors and acidification blockers [31] and that endocytosis pathway was crucial for co-stimulatory markers upregulation and IFN-α production [30]. Indeed, DENV-2 particles are detectable in pDC in the early stage (2 h) before viral degradation in lysosomes. However, after 18 h we did not detect viral antigens suggesting an absence of viral replication into pDC. Furthermore, lysosomal acidification impairment allowed detection of DENV-2 in pDC, contrasting with non-treated pDCs, suggesting that viruses are not disassembling. We did not observe an increase of non-structural protein 1 in culture supernatants (data not shown) after viral adsorption, supporting the incapacity for virus replication in pDCs. Our data is in accordance with others, as low levels of replicative negative strand RNA were found inside pDCs [30]. Therefore, we suggest that DENV-2 particle sensing occurs in endosomal compartments. Recognition but not infection of DENV-2 is responsible for IKpDC activation, whereas it leads to TRAIL relocalization and IFN-α production.

We next wonder whether IKpDC and IFN-α could inhibit DENV-2 replication in human monocytes, one of main target cells for DENV. Type I interferon have a crucial role during innate immune responses inhibiting viral replication and spreading of many viruses [68]. Binding and activation of IFN receptors triggers transcription of interferon stimulated genes, which induce products that are able to inhibit several steps of virus replication [69]. We found that DENV-2 infection was strongly diminished by treatment with IFN-α in human monocytes. In accordance with these data, other reports demonstrated that pre-treatment of several susceptible cell lines with type I interferon blocked DENV-2 replication through a protein kinase R (PKR)-dependent mechanism [70], [71], [72]. Indeed, recently, several interferon-stimulated genes such as interferon-inducible trans-membrane (IFITM) proteins were able to inhibit dengue infection in cell lines [73], [74]. However, type I interferon pathway is also subject to interference by many viruses that directly target pathways required for type I interferon response. Monocytes and monocyte-derived dendritic cells can produce IFN-α once they are infected by DENV, however at much lower levels compared to other viruses [66], [75], [76]. Moreover, several reports show degradation of downstream [77], [78], [79] and upstream [65], [80] interferon signaling pathways by DENV non-structural proteins. Although DENV blocks type I IFN pathway, the cytokine still remains protective for other uninfected cells reducing viral spreading during infection as described by others [81].

In our study, infected monocytes co-cultured with IKpDCs displayed a dramatic reduction in viral load that could be partially reversed by lysosomal blockage. Viral detection was negatively related to IFN-α detection in cocultures of monocytes and pDCs. IKpDC activation may play an important role for a rapid viral clearance. TRAIL has been reported as a potential antiviral factor for DENV replication [40]. Because TRAIL expression and production by monocytes is induced by IFN-α [37], we tested several concentrations of recombinant TRAIL on monocytes, and we confirmed the antiviral function as published before [40]. However, membrane TRAIL blockage on IKpDC had minimal effect on viral load or apoptosis during cocultures (data not shown). Moreover, IKpDC had no significant effect on DENV-2-infected monocyte apoptosis, suggesting that the anti-viral effect of pDC is mainly due to IFN-α and/or TRAIL on viral replication and not to cell death. Although, both TRAIL and IFN-α were fundamental in reducing viral load in HIV-infected CD4+ T cell/pDC co-cultures [82], [83]. For DENV, type I interferon was sufficient to largely reduce viral infection rates. Therefore, because we could not demonstrate that IKpDCs have a role in killing infected monocytes, this population may modify the outcome of the disease by producing massive quantities of IFN-α that would in turn block dengue replication in monocytes before adaptive immune responses ensues.

Finally, we showed in this work that DF patients harbored higher frequencies of circulating activated pDC and higher IFN-α/TRAIL levels compare to severe cases. DENV is activating pDC response in terms of IFN-α production and membrane TRAIL expression. We demonstrated that DENV mainly activates the endocytosis pathway and not the infection pathway, as we did not detect viral infection in pDC. Furthermore, our *in vitro* co-cultures data strongly support a crucial antiviral role for activated pDC and IFN-α by dramatically reducing viral spread. Even though, studies on DENV evasion from pDC response are still needed, we believe that pDC activation in patients' blood may contribute in the future to the establishment of good prognostic immune response together with clinical manifestations/warning signs.

## Supporting Information

Figure S1 **DENV-2 viral particles concentration by ultracentrifugation and infectivity assays on C6/36 mosquito cells.** C6/36 mosquito cell line was DENV-2 infected, supernatant were collected 10 days later and clarified by 1,000 g centrifugation. Cell-depleted supernatant was either stored (DENV-2 SNT) or ultracentrifugated at 100,000 g for 1 hour (DENV-2 UC) and stored (**A**). C6/36 cells were infected with equivalent dilutions (1–5 of SNT and 1–100 of UC) or not infected (mock) for 48 hours. (**B**) DENV envelope proteins (green) and DAPI-colored nucleus of C6/36 infected cells with equivalent dilutions of DENV-2 SNT and DENV-UC. White bar represents 25 µm. (**C**) DENV antigens detected by flow cytometry of C6/36 infected with DENV-2 SNT (orange) and DENV-2 UC (blue) 48 h after infection. (**D**) DENV antigens extra- and intracellular detection in DENV-2-UC-infected C6/36 cells after viral adsorption (0 h) or at 48 hours (48 h) of infection. Overlay histograms for DENV-2 UC (blue) and uninfected cells (grey). (**E**) DENV positive C6/36 according to time of infection. All data represents one out of two independent experiments.
(TIF)

Figure S2 **Monocytes and pDCs cocultures.** Freshly purified monocytes were infected with DENV-2 (MOI 10) for 48 hours, pre-treated or not with IFN-α. (**A**) IFN-α detection in DENV-2-infected pre-treated or not with IFN-α from three donors. (**B**) CD14+ purified monocytes were pre-treated or not with IFN-α, as pDCs were differently stimulated. After overnight incubation, monocytes were DENV-infected for 2 h, and then virus inoculum was removed. Whole pDCs cultures were added to infected monocytes during 48 h. DENV positive cells and IFN-α level were analyzed. (**C**) Isotipic DENV detection in CD14+ CD11c+ DENV-infected monocytes +CD14-CD11c-CD123+ DENV-activated pDCs after 48 h incubation. Data represents one out of two donors.
(TIF)

Figure S3 **Apoptosis assay on co-cultures of DENV-infected monocytes and pDCs.** Monocytes were infected with DENV-2 or mock and then co-cultured with or without CpG-stimulated with or without pDCs. After 48 hours of infection, cultures were collected and stained for AnnexinV (*y* axis) and TOPRO3 (*x* axis). Dot plots represent flow cytometry profiles for one representative donor.
(TIF)
