# Supplementary figures and images for: Dengue Virus Activates Membrane TRAIL Relocalization and IFN-α Production by Human Plasmacytoid Dendritic Cells In Vitro and In Vivo
Source: PLoS Negl Trop Dis. 2013 Jun 6;7(6):e2257. doi: 10.1371/journal.pntd.0002257 (PMC3675005; doi:10.1371/journal.pntd.0002257)

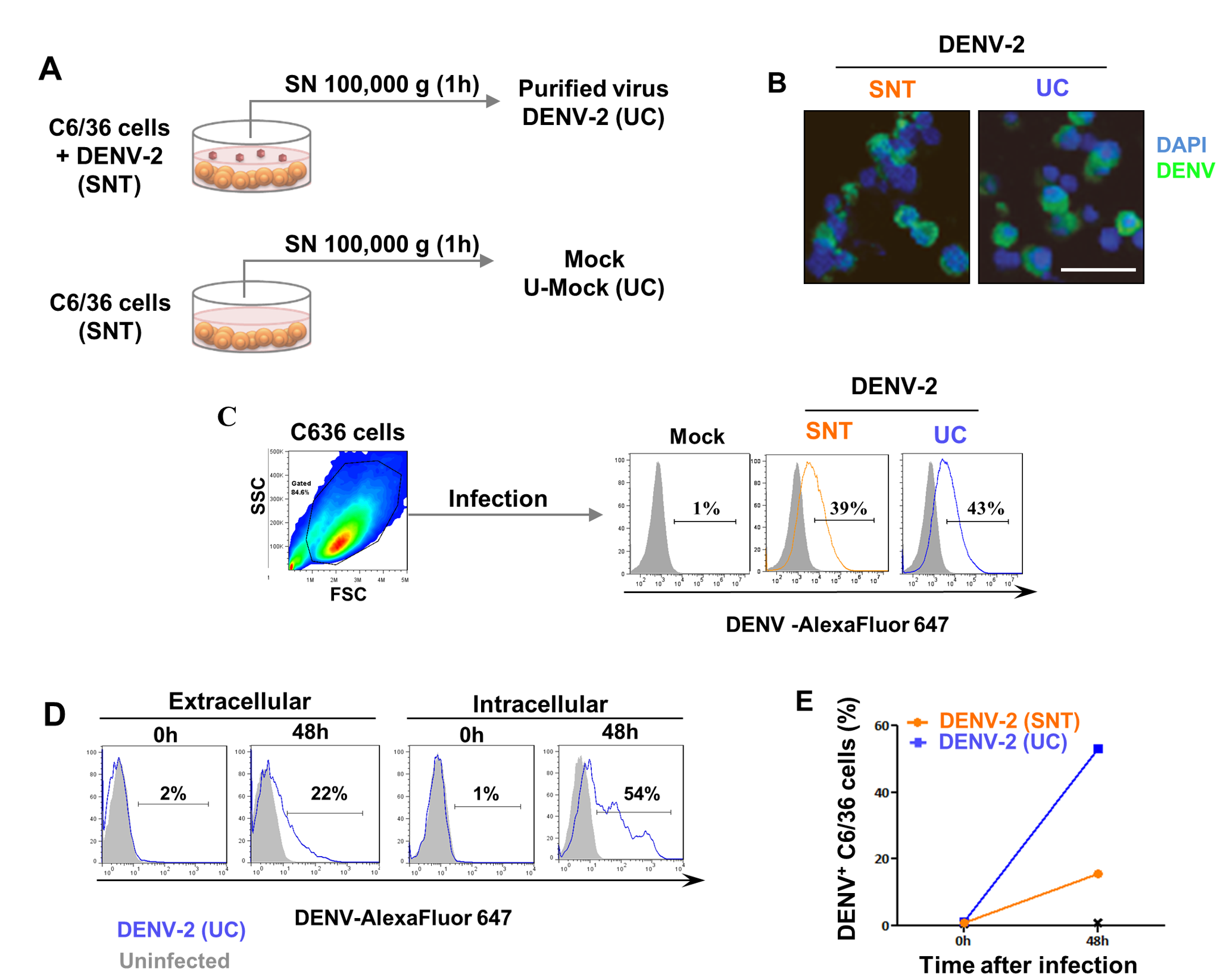

Supplement: Figure S1 — DENV-2 viral particles concentration by ultracentrifugation and infectivity assays on C6/36 mosquito cells. C6/36 mosquito cell line was DENV-2 infected, supernatant were collected 10 days later and clarified by 1,000 g centrifugation. Cell-depleted supernatant was either stored (DENV-2 SNT) or ultracentrifugated at 100,000 g for 1 hour (DENV-2 UC) and stored (A). C6/36 cells were infected with equivalent dilutions (1–5 of SNT and 1–100 of UC) or not infected (mock) for 48 hours. (B) DENV envelope proteins (green) and DAPI-colored nucleus of C6/36 infected cells with equivalent dilutions of DENV-2 SNT and DENV-UC. White bar represents 25 µm. (C) DENV antigens detected by flow cytometry of C6/36 infected with DENV-2 SNT (orange) and DENV-2 UC (blue) 48 h after infection. (D) DENV antigens extra- and intracellular detection in DENV-2-UC-infected C6/36 cells after viral adsorption (0 h) or at 48 hours (48 h) of infection. Overlay histograms for DENV-2 UC (blue) and uninfected cells (grey). (E) DENV positive C6/36 according to time of infection. All data represents one out of two independent experiments. (TIF) [file pntd.0002257.s001.tif]

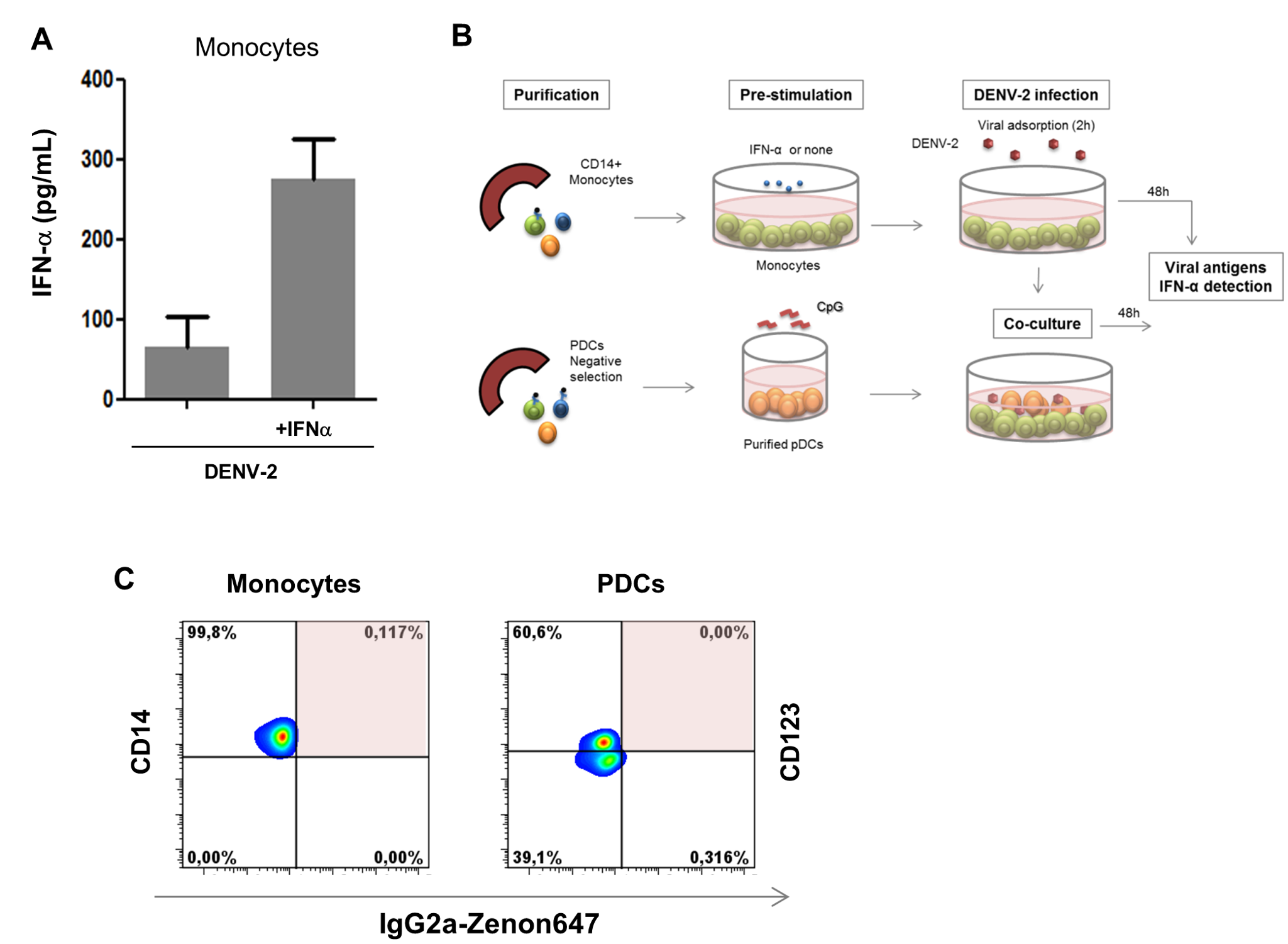

Supplement: Figure S2 — Monocytes and pDCs cocultures. Freshly purified monocytes were infected with DENV-2 (MOI 10) for 48 hours, pre-treated or not with IFN-α. (A) IFN-α detection in DENV-2-infected pre-treated or not with IFN-α from three donors. (B) CD14+ purified monocytes were pre-treated or not with IFN-α, as pDCs were differently stimulated. After overnight incubation, monocytes were DENV-infected for 2 h, and then virus inoculum was removed. Whole pDCs cultures were added to infected monocytes during 48 h. DENV positive cells and IFN-α level were analyzed. (C) Isotipic DENV detection in CD14+ CD11c+ DENV-infected monocytes +CD14-CD11c-CD123+ DENV-activated pDCs after 48 h incubation. Data represents one out of two donors. (TIF) [file pntd.0002257.s002.tif]

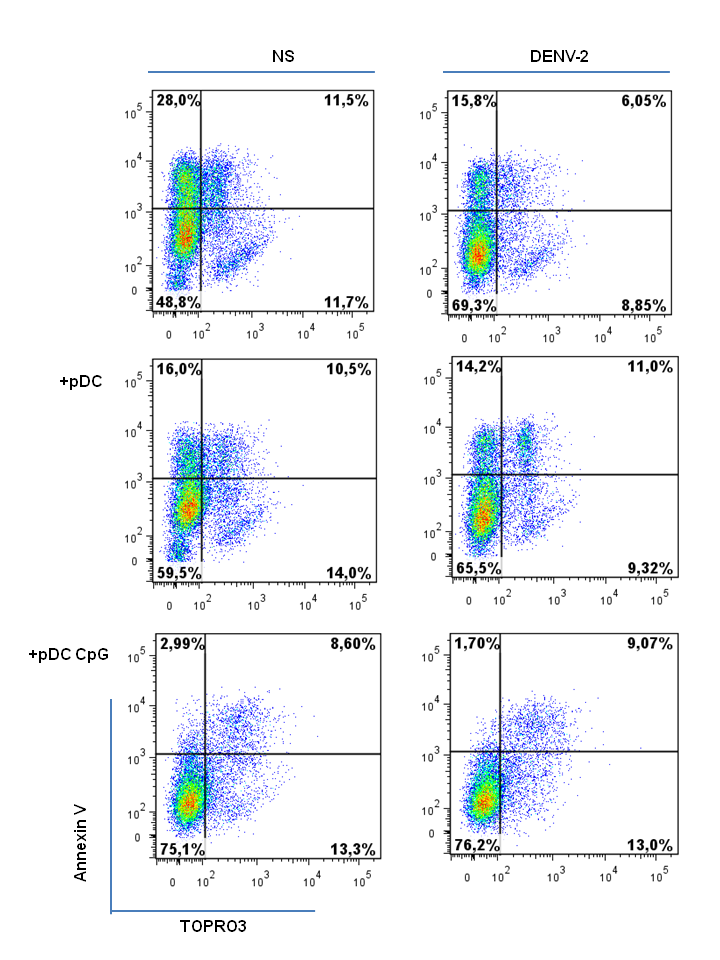

Supplement: Figure S3 — Apoptosis assay on co-cultures of DENV-infected monocytes and pDCs. Monocytes were infected with DENV-2 or mock and then co-cultured with or without CpG-stimulated with or without pDCs. After 48 hours of infection, cultures were collected and stained for AnnexinV (y axis) and TOPRO3 (x axis). Dot plots represent flow cytometry profiles for one representative donor. (TIF) [file pntd.0002257.s003.tif]
